# Vacuum curette lumbar discectomy mechanics for use in spine surgical training simulators

**DOI:** 10.1038/s41598-022-17512-5

**Published:** 2022-08-06

**Authors:** Trevor Cotter, Rosaire Mongrain, Mark Driscoll

**Affiliations:** 1 Musculoskeletal Biomechanics Research Lab, Department of Mechanical Engineering, McGill University, Montreal, H3A 0C4 Canada; 2 Orthopedic Research Laboratory, Montreal General Hospital, Montreal, QC H3H 1V8 Canada

**Keywords:** Mechanical engineering, Translational research

## Abstract

Simulation in surgical training is a growing field and this study aims to understand the force and torque experienced during lumbar spine surgery to design simulator haptic feedback. It was hypothesized that force and torque would differ among lumbar spine levels and the amount of tissue removed by ≥ 7%, which would be detectable to a user. Force and torque profiles were measured during vacuum curette insertion and torsion, respectively, in multiple spinal levels on two cadavers. Multiple tests per level were performed. Linear and torsional resistances of 2.1 ± 1.6 N/mm and 5.6 ± 4.3 N mm/°, respectively, were quantified. Statistically significant differences were found in linear and torsional resistances between all passes through disc tissue (both *p* = 0.001). Tool depth (*p* < 0.001) and lumbar level (*p* < 0.001) impacted torsional resistance while tool speed affected linear resistance (*p* = 0.022). Average differences in these statistically significant comparisons were ≥ 7% and therefore detectable to a surgeon. The aforementioned factors should be considered when developing haptic force and torque feedback, as they will add to the simulated lumbar discectomy realism. These data can additionally be used inform next generation tool design. Advances in training and tools may help improve future surgeon training.

## Introduction

Virtual-reality (VR) simulation training is an essential tool in training techniques for fields ranging from aviation to driving and beyond^1–5^. Simulators have been able to identify operator skill levels and are required in some cases before professionals are allowed to perform their duties^6–8^. Recent computational and simulation advances have enabled these tools to reach more industries, each with unique challenges. In medicine, the complex nature of the human body, as well as the risks associated with surgery, mean simulators have the potential to revolutionize the way surgeons prepare for procedures^9,10^.

### Surgical simulation

Surgery carries many dangers, thus the importance of effective surgeon practice and training is evident. Traditionally, surgeons have undergone a combination of classroom, animal, and cadaveric surgical training before approaching a living patient^2,11,12^. However, each of these training methods have limitations such as inaccurate anatomy or physiological response^2^. Alternatively, simulators with various degrees of complexity have been developed to train surgeons on a variety of procedures, from analog laparoscopic knot tying to full VR brain tumor resection^13,14^. The addition of robotic haptic, or touch, devices has enabled these simulators to become more adaptable by removing disposable components such as synthetic tissues and using robotic components to communicate the feeling of them consistently with minimal maintenance^2^. The demand for this type of training is evident by the numerous simulators that have been developed for spine surgery alone^2,15–18^. However, simulators using this technology may require tissue testing and an understanding of the biomechanics of the procedure.

Robotic haptic feedback must communicate the sensations a surgeon encounters during a procedure. This can be broken into two categories: the force and torque present in the procedure, and the force and torque felt by the surgeon.

### Tissue mechanics

Many techniques exist to mechanically characterize tissues. Properties such as elastic modulus and tensile strength can be derived from tests that measure force or torque and linear or angular displacement on a mechanical tester^19,20^. While this equipment is often limited to one or two axes, multiple tests can be used to generate a multi-axial characterization of the tissue^21,22^. Destructive tests often result in complex tissue mechanics. For needle insertion, existing studies have considered the total force $f_{needle}$ to be the sum of the tissue deformation ($f_{stiffness}$), friction ($f_{friction}$), and cutting ($f_{cutting}$) forces, which are dependent on position, $x$^23^:

1 $$f_{needle} \left( x \right) = f_{stiffness} \left( x \right) + f_{friction} \left( x \right) + f_{cutting} \left( x \right)$$

### Human perception of force

The mechanical understanding of biological tissues can be used to inform a robot to communicate the tissue force to a user. However, this communication must consider the boundaries of human perception. The just-noticeable difference (JND) is a measure of the minimum perceptible change in force. Hands and fingers are extremely sensitive to the sense of touch, and it has been found that intentional training can improve surgeon skills^24–26^. A JND of [5, 10]% change has been observed during [2, 10] N loads between fingers or in elbow extension, but can vary based on finger, training, and frequency^27–30^. Work on haptic devices has found higher JNDs, including 23 ± 13% and 34 ± 24% detectable changes for force and torque on the ranges of [0.4,8.8] N and [20,410] N mm, respectively^31^. While existing literature shows a wide range of observed JNDs, it is necessary to consider this sensitivity when determining how users will interpret differences in simulated tissue.

### Spinal mechanics studies

Spinal orthopedic procedures are of particular interest in new surgical simulators^2^. The spine is made up of alternating rigid vertebra and flexible intervertebral discs (IVDs) to create a flexible, supportive structure that protects the spinal cord, cauda equina and other anatomies. Over time or due to injury, IVDs can become compressed or deform, affecting the nerve root exiting from the cauda equina and causing intense pain^32–34^. Treatment of back pain is of immense importance, as it affects 80% of people at some point in their lives and is the leading cause of disability in the world^35,36^. Many treatment options for this pain exist, but the present work focuses on one surgical intervention: lumbar interbody fusion (LIF)^37^.

A LIF procedure aims to relieve pressure on the exiting nerve from the cauda equina. A surgeon performs a discectomy, or removal of the IVD. The surgeon then uses a curette to remove the nucleus pulposus and prepare the endplates of the IVD. With a gap now created, an interbody cage may be placed where the collapsed IVD had been. Bone graft is added at the interbody cage to fuse the two adjacent vertebrae at an appropriate spacing. Finally, pedicle screws and rods are placed to maintain the stability of the vertebrae during healing.

Like many other surgical procedures, modern developments have improved LIF, yielding minimally invasive (MI) procedures. MI surgeries have been found to decrease hospital stays and recovery times^37–39^. As such, a new vacuum curette was developed (Concorde Clear MIS Discectomy Device; DePuy Synthes; Boston, USA) to allow surgeons to perform discectomies with a single tool faster and safer than existing curettes^40^. The adoption of a novel tool requires training. In a surgical simulator context, this also requires the characterization of force and torque encountered when using this tool.

Biomechanical studies performed on the IVD have traditionally been focused on various loading scenarios a person may encounter^41–44^. Few studies deal with the force as surgeons encounter them during surgery^45^. While the known biomechanical properties of the IVD may predict spine behavior under loading, they may be inadequate in predicting mechanical interactions with surgical tools. The unique shape, cutting surfaces, and vacuuming effect of the Concorde Clear may yield unpredictable biomechanical responses. As a result, this work compares peak force and torque, which are the aggregate of stiffness, friction, cutting, and other factors during tool insertion as shown in Eq. (1). Similarly, resistance for each of these quantities is defined by the change of each quantity during loading. Therefore, linear resistance is N/mm and torsional resistance is N mm/°.

This manuscript hypothesizes that discectomy force and torque will be dependent on spinal level and removed tissue at a ≥ 7% difference, a magnitude detectable to a surgeon. This data can then be used to inform haptic feedback in relevant surgical simulators while drawing attention to the importance of such model selection in other biomechanical studies of the disc.

## Materials and methods

Mechanical testing was performed on human tissues to characterize linear and torsional movements. These data were then analyzed and compared to understand how they differ between anatomical and procedural conditions.

### Sample preparation

Two fresh frozen cadaveric torsos were acquired from Science Care, Inc. (Phoenix, USA) and tested with ethical approval (McGill University Faculty of Medicine Institutional Review Board (IRB) A04-M13-18A) in accordance with all relevant guidelines, regulations, and consent requirements. Both samples had no history of radiation treatment or spinal surgery. X-rays were used to measure the height of each lumbar IVD to indicate IVD degeneration. Specifications for the cadavers can be seen in Table 1. All IVD height measurements were taken from El-Monajjed et. al, who used the same specimens and methods for preparation^45^. Cadavers were stored at − 20°C, thawed for 5 days at 2°C, and held at room temperature for 72–96 hours before testing. A 30 × 30 cm posterolateral window was removed from the skin, fascia, and muscle to expose the posterior lumbar spine. A 1 × 1 cm annulotomy was performed posterolaterally to gain access to the IVD as would be done in an MI LIF.

**Table 1:** Cadaveric torso properties.

Cadaveric torso properties					
Cadaver				C1	C2
Gender				M	M
Age				63	69
Height			cm	175	178
Weight			kg	73	86
Collapsed Disc				None	L_4_L_5_
IVD Dimensions	L_1_L_2_	Height	mm	10.0	5.2
		Width	mm	44.7	49.4
	L_2_L_3_	Height	mm	10.3	7.6
		Width	mm	46.1	45.7
	L_3_L_4_	Height	mm	11.6	5.7
		Width	mm	47.6	60.6
	L_4_L_5_	Height	mm	10.3	3.1
		Width	mm	54.1	NA

The cadaver measurements are from a previous work^45^. IVD width is the lateral width.

### Testing setup

**Figure 1:**
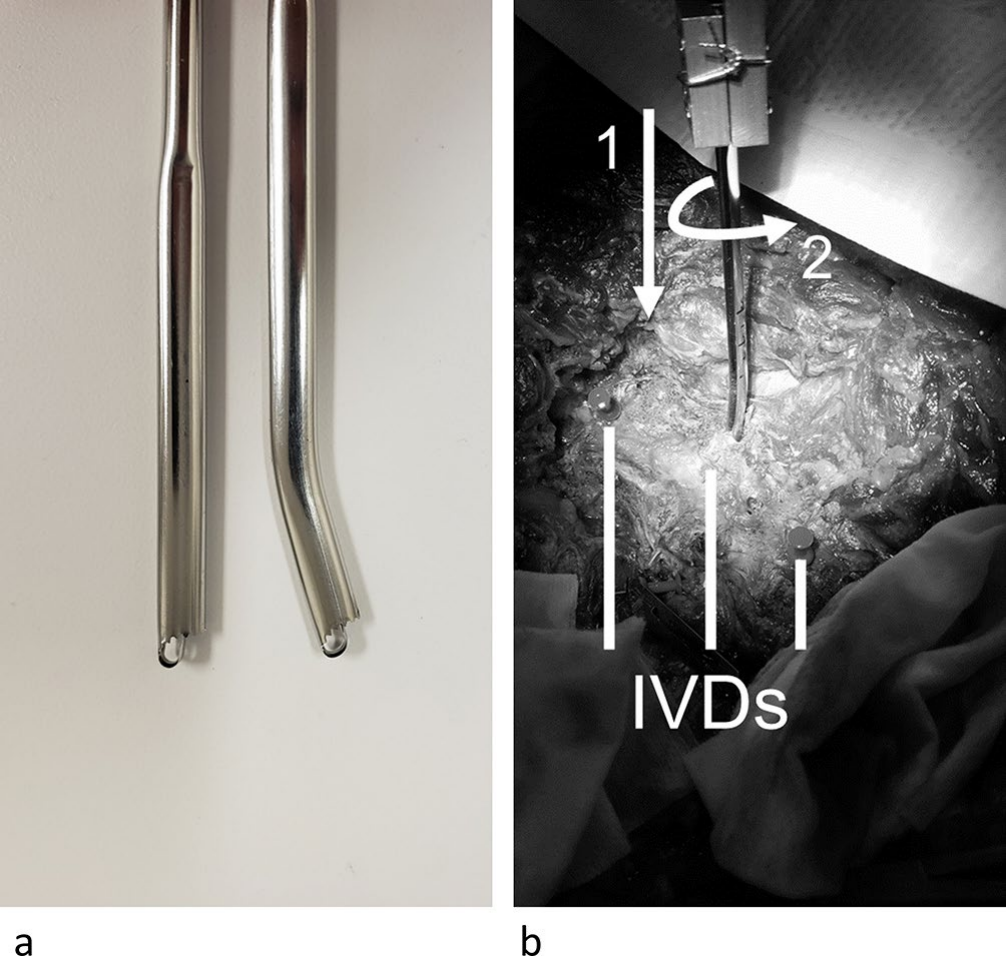
Concorde Clear tools used in the test. (**a**) shows the straightened tool (left) used in the linear test as well as the normal bent tool (right) used in the torsional test. (**b**) shows the test setup and motion, where the tool is inserted (1) into the intervertebral disc (IVD) and twisted (2). The linear test involved motions 1 and 2, while the torsional test was only motion 2. IVDs are marked.

A custom jig supported the cadaveric torso. The torso was laid on its chest and rotated to allow a linear tester to penetrate at approximately 40° lateral to a fully posterior approach. Testing was performed with an MTS 858 Mini-Bionix II testing apparatus and a force and torque load cell of 2.5 kN and 25 N m, respectively (662.20D-01, MTS Systems Corporation; Minneapolis, USA). Custom fixturing connected the tester to one of two tools (Fig. 1).

### Linear testing

**Figure 2:**
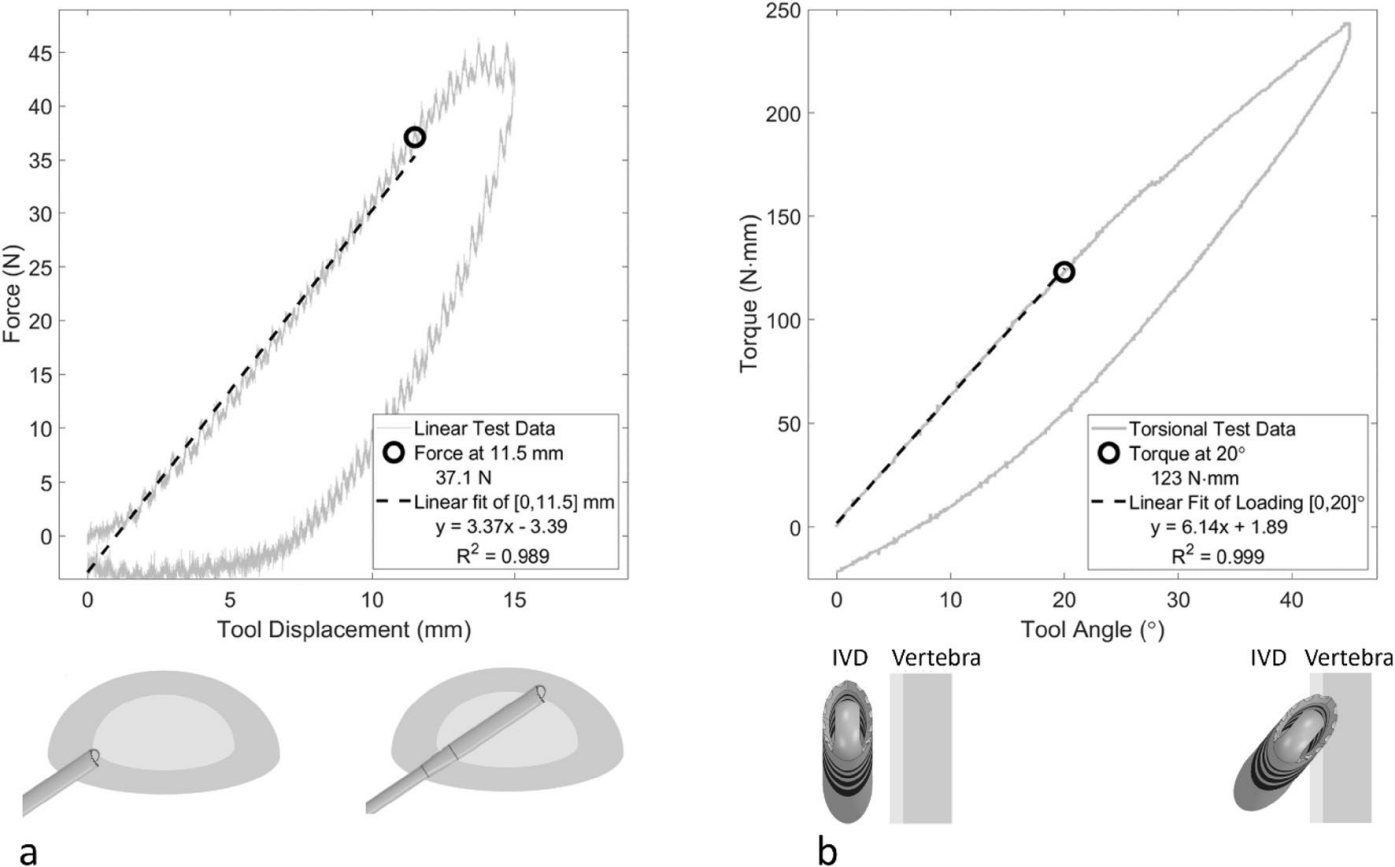
Examples of linear (**a**) and torsional (**b**) tests with extracted peak and resistance values for the first trial of the right side of C2 L_3_L_4_. The torsional test was performed at a 5 mm depth. Schematics of the tool orientation within the intervertebral disc (IVD) are shown.

The first test was a linear insertion that mimicked a surgeon penetrating the IVD. The load cell was secured to a straightened version of the Concorde Clear shaft tip, shown in Fig. 1a. The shaft tip was lowered to 5 mm inside the IVD space before beginning. The shaft tip was then inserted at a rate of 0.25 mm/s from [12–15] mm of tool travel, depending on disc size. Meanwhile, the tool was rotated [± 20]° at 20°/s to prevent snagging and ensure penetration into the IVD. The tool was then withdrawn at the same rates until it returned to its starting position. The tool path can be seen in Fig. 1b, where motions 1 and 2 were performed. Time, position, force, angle, and torque were all recorded at 100 Hz. An example of the position and force results can be seen in Fig. 2a. The test was performed 3 times on the left and right sides of lumbar IVDs between L_1_ and L_5_. Additional speed studies were also performed. Linear speeds of 0.25, 0.50, 0.75, and 1.00 mm/s were compared on the right side of C1 L_3_L_4_. Torsional speeds of 10, 20, 30, and 40°/s were compared on the right side of C1 L_4_L_5_. Test details are shown in Table 2.

**Table 2:** Testing parameters.

Testing parameters			
Test name		Linear test	Torsional test
IVD levels		L_1_L_2_–L_4_L_5_	
Sides		Left and Right	
Linear motion	Waveform	Triangle	NA
	Starting position	5 mm inside IVD	NA
	Range	0–12/15 mm	NA
	Speed	0.25 mm/s	NA
	Additional speed tests	0.50, 0.75, 1.00 mm/s	NA
Torsional motion	Waveform	Sinusoidal	Sinusoidal
	Starting position	0°	0°
	Range	± 20°	± 45°
	Speed	40°/s	2°/s
	Additional speed tests	20, 60, 80°/s	3, 4, 6, 8°/s
Number of Trials		3	5

### Torsion testing

The second test was a torsional motion that mimicked a surgeon twisting inside the IVD space. The load cell was secured as for the linear test but with an angled Concorde Clear shaft tip, shown in Fig. 1a. The shaft tip was lowered to 5 mm inside the IVD space and twisted at 2°/s to [± 45]° for a total of 5 full cycles. One cycle represented a tool path that proceeded through angles of 0°, 45°, − 45°, and finally 0°. The tool path can be seen in Fig. 1b, where only motion 2 was performed. Data recording was the same as the linear testing. An example of the angle and torque results can be seen in Fig. 2b. This test was repeated at a 20 mm penetration depth on the right and left sides of each lumbar IVD between L_1_ and L_5_. Torsional speeds of 2, 3, 4, 6, and 8°/s were compared on the right side of C1 L_4_L_5_. Test details are shown in Table 2.

### Data analysis

The data were then analyzed for comparison. Initial position, angle, force, and torque were normalized at the start of each test. This applied to all linear tests, but notably only the first torsional test. After extracting the relevant parameters, a variety of statistical comparisons were performed to determine the significance of differences between cadavers (C1, C2), lumbar levels (L_1_L_2_, L_2_L_3_, L_3_L_4_, L_4_L_5_), and tool passes (1, 2, 3, (4, 5)) of linear (0.25, 0.50, 0.75, 1.00 mm/s) and torsional speed (10, 20, 30, 40°/s). Additional comparisons of penetration depth (5, 20 mm) and torsional speed (2, 3, 4, 6, 8°/s) were performed on the torsional tests. A Mann–Whitney U test was used to compare two conditions, Kruskal–Wallis was used for tests with three or more conditions, and data were compared to IVD height using Spearman correlation after confirming they were not normally distributed using a Shapiro–Wilk normality test^46–49^. Comparisons are summarized in Table 3, where bold, italicized values have a significance of α ≤ 0.05.

**Table 3:** Statistical comparisons of testing conditions.

Statistical comparison of testing conditions					
**Linear tests**					
Comparison	Cadavers	Lumbar level	Pass number	Linear speed	Torsional speed
Force peak					
All passes	1.46E−01	7.23E−02	***2.05E−03***	***1.56E−02***	***2.16E−02***
Pass 1	5.74E−01	1.35E−01	–	–	–
Pass 2–3	***2.05E−02***	1.52E−01	*3.00E−01	8.33E−02	1.04E−01
Test method	Mann–Whitney U	Kruskal–Wallis			
Linear resistance					
All passes	2.36E−01	1.58E−01	***1.49E−03***	***2.16E−02***	***1.88E−02***
Pass 1	4.42E−01	1.13E−01	–	–	–
Pass 2–3	***3.32E−02***	2.49E−01	*3.76E−01	8.33E−02	8.33E−02
Test method	Mann–Whitney U	Kruskal–Wallis			

**Torsion tests**					
Comparison	Cadavers	Depth	Lumbar level	Pass number	Torsional speed
Torque peak					
All passes	***1.17E−05***	***5.69E−15***	***2.11E−10***	***1.63E−02***	***1.19E−03***
Pass 1	***2.13E−02***	***1.49E−05***	***4.71E−02***	–	2.12E−01
Pass 2–5	***1.93E−04***	***2.24E−11***	***2.62E−09***	2.56E−01	***1.04E−02***
Test method	Mann–Whitney U		Kruskal–Wallis		
Torsional resistance					
All passes	***1.29E−04***	***8.78E−05***	***5.27E−09***	***1.42E−03***	5.79E−02
Pass 1	9.20E−02	***2.69E−03***	***3.74E−02***	–	6.82E−01
Pass 2–5	***2.09E−04***	***4.34E−03***	***1.04E−07***	6.42E−01	8.29E−02
Test method	Mann–Whitney U		Kruskal–Wallis		

All bolded, italicized values have significance p ≤ 0.05 and exceed the JND threshold of 7%.

*Performed with Mann–Whitney U Test.

For linear tests, 11.5 mm of tool travel after the initial set position of 5 mm inside the IVD was used. A linear fit was performed on force versus position for the range of [0,11.5] mm, and the peak force at 11.5 ± 0.25 mm was extracted. A sample fit is shown in Fig. 2a.

For torsional tests, the first ± 20° of tool rotation was used. A linear fit was performed on torque versus angle for the range of [0, ± 20]° and the peak torque at ± 20 ± 0.2° (± 10 data points at 100 Hz) was extracted. A sample fit is shown in Fig. 2b.

## Results

Data and overall comparisons were considered separately with force in the linear tests and torque in the torsional tests.

### Force

#### Peak

**Figure 3:**
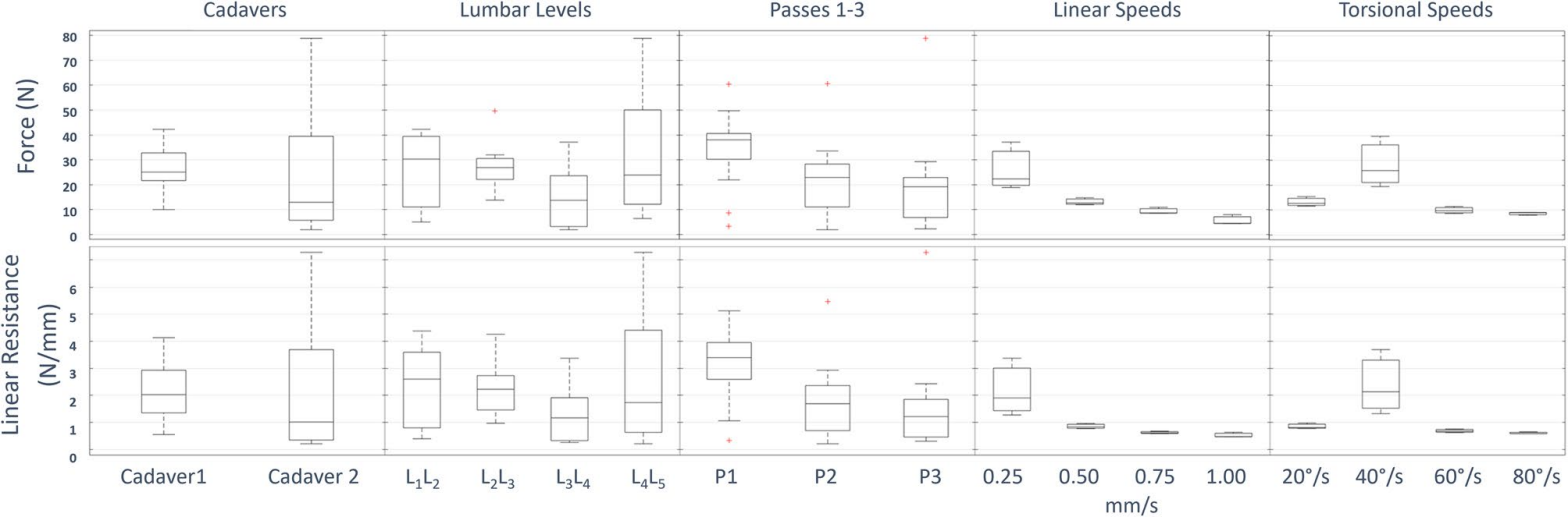
Force peak and linear resistance comparisons.

Peak force samples can be seen for multiple conditions in Fig. 3 Average peak force at 11.5 mm was 25.2 ± 16.7 N. There was a statistically significant difference between passes (P1, P2, P3). However, after the initial pass (P1), later passes (P2, P3) were similar, indicating that the results stabilize after the initial destructive pass (P1). Additionally, after the initial pass (P1) there was a statistically significant difference between cadavers (C1, C2). There was a significant difference when performing the test at linear speeds (0.25, 0.50, 0.75, 1.00 mm/s) and torsional speeds (10, 20, 30, 40°/s) when considering all trials. Boxplots containing this information are contained in Fig. 3, with corresponding *p* values in Table 3.

### Resistance

The linear resistance can be seen for multiple conditions in Fig. 3. Average resistance over the range [0,11.5] mm was 2.1 ± 1.6 N/mm. All statistical differences match those observed for peak values. Boxplots containing this information are contained in Fig. 3, with corresponding *p* values in Table 3.

### Disc height correlations

**Figure 4:**
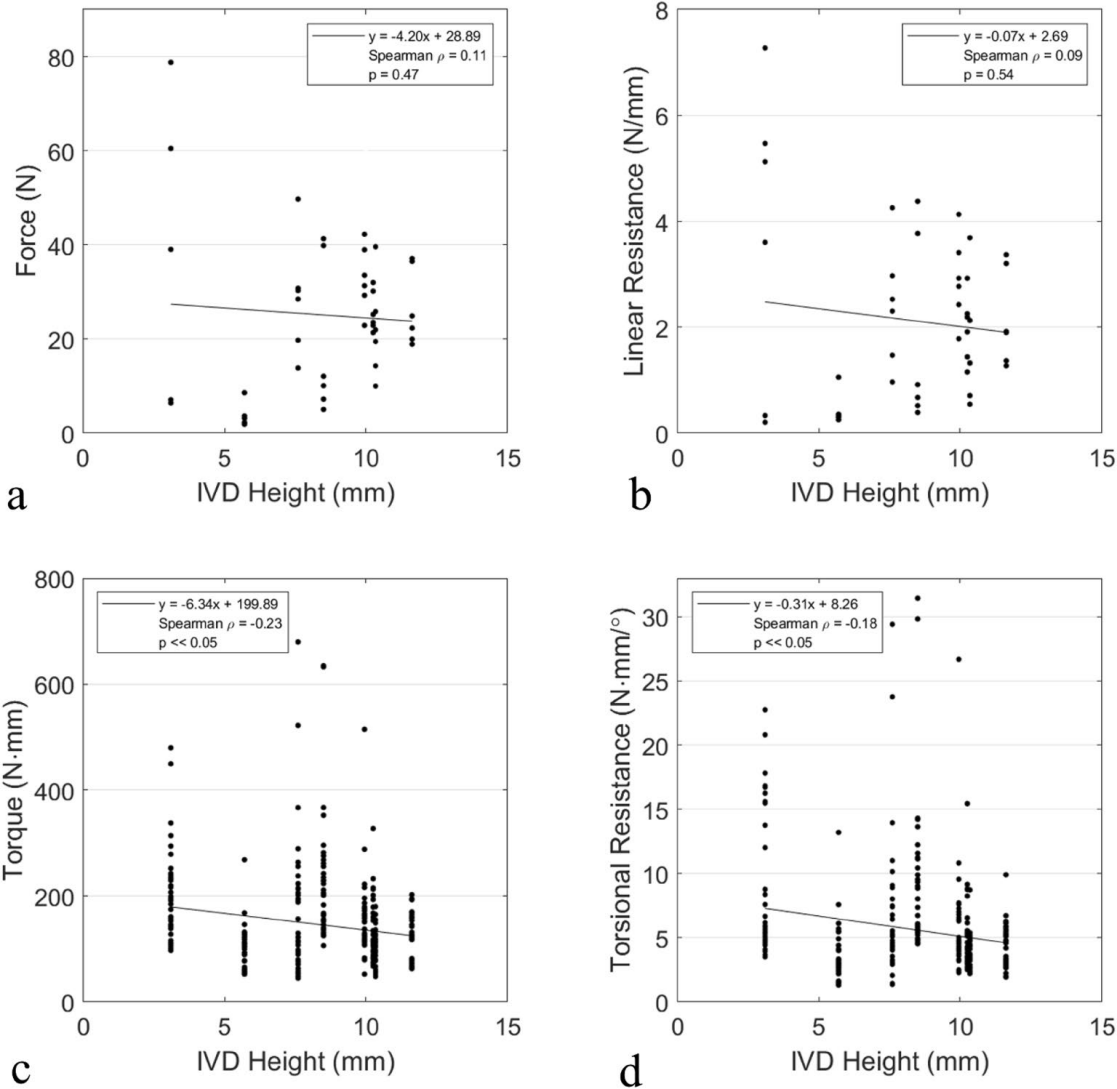
Linear (**a**,**b**) and torsional (**c**,**d**) peak and resistance values as correlated to intervertebral disc (IVD) height.

IVD height appeared to have no statistically significant correlation with either the peak or linear resistance as seen in Fig. 4a,b.

### Torque

#### Peak

**Figure 5:**
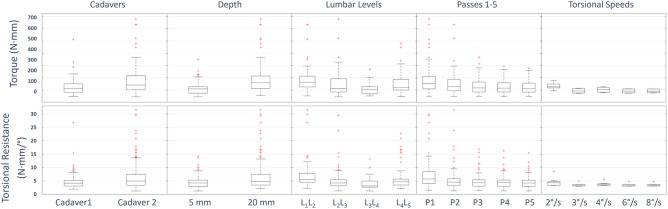
Torque peak and torsional resistance comparisons.

Peak torque samples can be seen in Fig. 5. Average torque magnitude at ± 20° was 146.6 ± 90.0 N mm. Statistically significant differences were observed between cadavers (C1, C2), depths (5, 20 mm), lumbar levels (L_1_L_2_, L_2_L_3_, L_3_L_4_, L_4_L_5_), and passes (P1, P2, P3, P4, P5). For torsional speeds (2, 3, 4, 6, 8°/s), a difference was observed across all passes and after the initial pass (P1), but not for the initial pass (P1). Boxplots containing this information are shown in Fig. 5, with corresponding *p* values in Table 3.

### Resistance

The torsional resistance can be seen in Fig. 5. The average resistance on the range [0,20]° was 5.6 ± 4.3 N mm/°. All statistical differences matched those observed for the peak values, with the exceptions that differences between torsional speeds (2, 3, 4, 6, 8°/s) for any pass combination and the initial pass (P1) between cadavers (C1, C2) were not significant. Boxplots containing this information are contained in Fig. 5, with corresponding *p* values in Table 3.

### Disc height correlations

IVD height appeared to have no statistically significant correlation with either the peak or torsional resistance, as seen in Fig. 4c,d.

## Discussion

The data presented show the range of expected force and torque present during a lumbar discectomy using the Concorde Clear. The hypothesis that force and torque would be dependent on spinal level and removed tissue at a ≥ 7% difference was confirmed in most cases. The difference between peak force and torque, as well as linear and torsional resistance, differed by ≥ 7% between initial and later passes through the tissue. Similar differences were found between lumbar levels, except for the peak force comparisons that did not meet the 7% threshold. To create a simulator or inform next generation discectomy tools, it is essential to identify how these data can be used to replicate or facilitate the surgical experience. This discussion focuses on distinguishing between anatomies or procedural conditions during the discectomy with respect to the JND of the user.

### Force

Peak force at 11.5 mm and linear resistance over [0,11.5] mm followed the same statistical patterns. The largest statistically significant difference across all conditions was between all passes (P1, P2, P3), with peak force decreasing after the initial pass (P1). This change suggests that the removal of tissue, or ${f}_{cutting}$ in Eq. (1), was an essential distinguishing element during the procedure. However, after the initial pass (P1), the measured force for later passes (P2, P3) was the same. Variability in the initial pass (P1) was large enough that the difference between cadavers (C1, C2) was insignificant. However, after this destructive initial pass (P1), the cadaveric variation (C1, C2) became visible. Additionally, both linear and torsional speeds significantly impacted the force of insertion. In contrast, IVD height, which was used as an indicator of IVD degeneration, did not show a correlation with a change in force or linear resistance. This disconnect may be attributed to anatomical geometry. IVD height inside the disc was larger than around its perimeter, meaning the tool did not need to separate adjacent vertebrae away to penetrate deeper tissue after it had already penetrated the disc. Therefore, it can be considered that the most important factors to consider when designing a simulator for insertion of the Concorde Clear are the number of times the tool has passed through the disc and the speed at which the device is being pushed and twisted.

### Torque

Peak torque at ± 20° and torsional resistance over [0, ± 20]° showed more statistical differences across comparisons than peak force and linear resistance. The peak torque was different between cadavers (C1, C2), penetration depths (5, 20 mm), and between all passes (P1, P2, P3, P4, P5), only being the same for the initial pass (P1) across the torsional speed (2, 3, 4, 6, 8°/s) tests. The torsional resistance was different across torsional speeds (2, 3, 4, 6, 8°/s) for all passes (P1, P2, P3, P4, P5), as well as the initial pass (P1) between cadavers (C1, C2). This suggests once again that the cutting torque, or other torque component only present in the initial pass, impacts the total torque significantly. Like the linear testing, both peak torque and torsional resistance were independent of IVD height and corresponding degeneration. While the tool did not need to further spread the IVD when penetrating, as in the force test, it did abut the vertebral bodies during rotation. Adjacent IVDs may have accommodated this distraction by compressing and absorbing the torque, leading to similar results for all IVDs. However, the collapsed IVD (C2 L_4_L_5_), was included in this data set, and yet there was still no observed correlation. Greater variability between test conditions other than IVD height such as cadaver, pass number, and speed implies that more factors must be considered when designing torque output in a simulator than when designing the force output.

### Simulator application

The data shown could be used to determine the appropriate force and torque needed in a Concorde Clear discectomy simulator. However, it was still necessary to determine if a user could distinguish between the statistically different conditions outlined above and in Table 3. Using a JND of 7%, as previously suggested, all statistically significant differences in peak force, peak torque, linear resistance, and torsional resistance shown in Table 3 would be detected by the surgeon. This implies that cadaveric differences, pass number, linear speed, and torsional speed should all be considered when determining the robotic force output, while lumbar level should not. Furthermore, cadaveric differences, tool depth, lumbar level, pass number, and torsional speed in some circumstances should be accounted for when determining the robotic torque output.

### Limitations

As with any study, there were limitations to its scope. One key shortcoming of this work was that only the tool travel was measured, not the displacement of the body. This means that for a given tool displacement, the actual penetration of the tool into the IVD was less. The cadaveric torso was intentionally allowed to move slightly within its jig to replicate the compression or movement a surgeon may experience during surgery. This setup introduced more variability into the study, as the specific orientation and support of the sample will have an impact on the test. This restricted the applicability of the study to determine material properties of the IVD but was necessary to replicate surgical conditions. This is why the peak and resistance values for both force and torque were used, as well as the aggregated subcomponents of each as shown in Eq. (1) and previous work^23^. Another limitation was the impact of IVD height on beginning the discectomy. Both versions of the Concorde Clear were inserted 5 mm into the disc space before beginning the linear or torsional tests, meaning that the difficulty of entering the IVD before removing tissue was not measured. Because all data were normalized at 5 mm penetration for the linear test, differences in this initial force to penetrate the IVD, which may be more difficult for short IVDs, were not considered. Similarly, the torsional tests were only normalized at the beginning of the cyclical testing and the act of normalizing these data for each loading cycle for each pass and direction could have affected the results. Using more samples could have prevent wear and tissue destruction from impacting the results when performing multiple tests on the same IVD, however, this is why the total sum of forces was considered in the study. Finally, the assumptions used here, of a 7% JND, were based on existing work. However, studies have also found that providing feedback, training, and frequency changes can have an impact on JND^30^. Additionally, visual feedback has also been shown to impact user JND^50^. It is possible that surgeons, through their extensive training, have developed greater sensitivity. This could be tested in the future in a manner comparable to previous work that found surgeon forces differed based on experience level^51^.

The force and torque profiles shown here can be used to inform a haptic robot to give feedback to a user or future tool design. Operator speed, number of passes, and patient differences should be considered when determining appropriate force and torque output. Lumbar level and tool depth should additionally be considered for proper torque output. By making these adjustments before and during the procedure, a simulator can be created to accurately mimic an MI LIF discectomy.

## Conclusions and future work

This work presents the first biomechanical study of MI discectomy using the Concorde Clear. A framework is provided for the measured force and torque, how they vary over time and between multiple conditions. Improvements in these measurements could be made by quantifying the amount of tissue removed and correlating it to the measured mechanics. This would enable better modeling for the force output by allowing the simulator to respond to tissue removal as the user proceeds through a procedure. Following simulator development, studies must be performed with surgeons to evaluate how experts perceive the mechanics that have been measured and subsequently integrated. Perhaps surgeon JND differs from that of the normal population and therefore the simulator must be sensitive to minute differences between tissues in the procedure. This would validate the study results by showing how effective these data are in a simulator that is both biomechanically accurate and relevant for training. Overall, this study provides a better understanding of the force and torque encountered by a surgeon using a tool, such as the Concorde Clear, during a lumbar discectomy, and how these measures can be applied in a simulated environment.

## Data Availability

The datasets used and analysed during the current study are available from M.D. on reasonable request.
